# Patrick Ruthven-Murray: Erfolgreich zum Medizinstudium – Wie ich mir einen Studienplatz in Deutschland oder im Ausland sichere. 2. überarbeitete und erweiterte Auflage

**DOI:** 10.3205/zma001090

**Published:** 2017-05-15

**Authors:** Michaela Zupanic, Marzellus Hofmann

**Affiliations:** 1 University of Witten/Herdecke, Witten, Germany; 2 University of Witten/Herdecke, Faculty of Health, Witten, Germany

## Recension

Four years after the first edition of his book "Erfolgreich zum Medizinstudium", the experienced academic advisor Patrick Ruthven-Murray presents his practical handbook on applying to medical school in a revised and extended edition.

Already in the preface, the author explicitly addresses his young audience, to whom he would like to offer a decision-making aid with clear words. Having clarified the right motivation, repectively counter-review of good versus bad reasons for the final degree doctor, the personal prerequisites for a good physician are named: indispensable willingness to perform, scientific interests, mental stability and social skills. This is followed by a very brief outline of medical education from undergraduate studies to residency training, whereby the author has not revised his reservations towards reform and model courses of study, assuming that students in these settings might be treated as "guinea pigs".

The application procedure for medical training at a public German university via hochschulstart.de is presented in a short and comprehensible manner and clarified by means of a timetable. Regarding the university-specific selection processes of all 35 medical faculties in Germany, the information on selection criteria, updated up to the post-decimal point, is systematically combined. With the chart for strategically setting the location preference, the reader can draft a first personal conclusion about her/his own chances for acceptance. As a new trend in the selection process, multiple mini-interviews (MMI), used in a few universities and private educational institutions, are described in detail with various objectives and tasks as well as solutions for standard situations. In his assessment of the various options the author expresses a clear-cut opinion on different approaches such as: "Does it make sense to wait? No!" He gives general advice for the preparation and recommendations for the application, also for applicants with rather small chances.

In the description of the possible alternative pathways to medical schools, correct and updated information is prepared. The mini-glossary, that had been erratic in the first edition, has been deleted without substitute, as it lacks any foundation in academic reality. The access requirements for studying at a private university and the now established Medical Schools are briefly explained in tabular form. In accordance with his recommendation, the author systematically documents all essential requirements and information on studying in Europe, since all degrees in the EU are recognized as equivalent. Unfortunately, the given hyperlinks to the application procedures of the universities are partially not working whilst the date of last query is missing.

As a conclusion, it can be stated that the handbook provides a clearly arranged and useful decision-making aid. The update for the summer semester 2017 briefly and precisely summarizes the necessary information for a desired application to medical schools in Germany or Europe. The fact that everyone can begin a medical education promptly, after reading this practical guide, no longer seems questionable.

## Competing interests

The authors declare that they have no competing interests. 

